# 3D‐Printed Electroanatomic Twins of Cadaveric Cochleae: A Platform for Cochlear Implant Testing

**DOI:** 10.1002/lio2.70509

**Published:** 2026-07-17

**Authors:** Chloe Swords, Iwan Vaughan Roberts, Sita Tarini Clark, Filip Hrncirik, Botian Huang, François Guérit, Cecilia Brassett, Manohar L. Bance

**Affiliations:** ^1^ Department of Physiology, Development and Neuroscience University of Cambridge Cambridge UK; ^2^ Department of Clinical Neurosciences University of Cambridge Cambridge UK; ^3^ Cambridge Hearing Group, MRC Cognition & Brain Sciences Unit University of Cambridge Cambridge UK

**Keywords:** 3D printing, additive manufacturing, auditory system, cochlear implants, electrodes, hearing loss

## Abstract

**Objectives:**

Cochlear implants (CIs) can restore hearing to individuals with severe deafness, yet clinical outcomes vary widely because it remains difficult to predict how electrical currents interact with the unique anatomy of each cochlea. A central challenge in auditory rehabilitation is the lack of experimental systems that capture both the anatomical fidelity and the electrical properties of the human cochlea. To address this need, we developed electroanatomic twins: three‐dimensional, 3D printed models of human cochleae that incorporate anatomically accurate features of the otic capsule and conductive biomimetic structures designed to reproduce tissue resistivity.

**Methods:**

Electroanatomic cochlea twins were generated from high resolution microCT scans of cadaveric specimens and from clinical CT scans, and they allowed reproducible, high‐resolution mapping of voltage fields during CI stimulation.

**Results:**

These models reproduced intraoperative spread of current profiles with a root mean square error below 0.1 kΩ, closely matched cadaveric impedance spectra, and recapitulated spatial voltage distributions under monopolar, bipolar, and tripolar stimulation.

**Conclusion:**

By combining anatomical precision with electrical realism, electroanatomic twins provide a robust translational platform for systematic testing of electrode designs and programming strategies. This approach has the potential to guide individualized CI programming and thereby improve auditory outcomes, while also establishing a generalisable framework for modeling electroanatomic interactions that could accelerate the development of personalized bioelectronic therapies.

**Level of Evidence:**

Level 3.

## 1 Introduction

Cochlear implants (CIs) restore hearing in individuals with severe sensorineural hearing loss by electrically stimulating the auditory nerve, bypassing damaged hair cells. Despite over one million recipients worldwide [1], outcomes vary widely, in part because electrical currents spread beyond targeted neural populations, reducing spectral resolution and impairing speech perception [2, 3, 4, 5]. Contributing factors include the electrical conductivity of cochlear fluids and tissues, as well as individual anatomical variability in cochlear morphology [6, 7, 8].

Modern CI devices stimulate cochlear regions in an interleaved pattern to limit interaction between channels [9, 10]. Standard monopolar (MP) stimulation, with the return electrode located outside the cochlea, produces broad current spread that can reduce spectral selectivity. Focused configurations (bipolar (BP), partial tripolar (pTP), and tripolar (TP)) position source and return electrodes closer together to constrain current spread. While some studies report improved speech perception with these strategies [11, 12], others show mixed or inconclusive results [13, 14], highlighting the need for a better understanding of how electrode configuration and cochlear anatomy interact to shape intracochlear electrical fields.

Understanding the way in which electrical current is distributed within human cochleae is therefore critical for optimizing CI performance. Finite element models (FEMs) provide insights into intracochlear bioelectric behavior [15, 16, 17], but they often struggle to capture complex electrode–cochlea interactions, nonlinearities, and transient electrical phenomena, and are computationally intensive [18, 19]. Physical models provide a complementary approach, enabling real‐world testing of electrode designs and stimulation strategies under controlled yet anatomically realistic conditions [19, 20]. However, systematic validation of these 3D‐printed models against cadaveric measurements with detailed waveform and complex impedance analyses remains lacking. Such analyses can reveal subtle markers of tissue or anatomical variation, informing strategies for targeted neural stimulation [21].

This study developed and validated a 3D‐printed, anatomically precise human cochlea model to investigate current spread during cochlear implant stimulation. Cochlear anatomy was derived from a high‐resolution template and integrated into a semi‐automated workflow for analysis of cochlear size and shape in both clinical CTs and microCTs of cadaveric specimens. Validation included SCINSEVs (Stimulation‐Current‐Induced Non‐Stimulating Electrode Voltages), complex impedance measurements, and micro‐electrode recordings within the modiolus, comparing 3D‐printed models to cadaveric cochleae and intraoperative recordings. This platform was used to systematically examine the effects of array type, current focusing strategies, and anatomical variation on electric field spread, providing new insights into spatial selectivity and potential strategies to optimize cochlear implant outcomes.

## 2 Results

### 2.1 Experiment 1: Development of an Electro‐Mimetic 3D‐Printed Model Validated Against Clinically Measurable Voltage Data

**FIGURE 1:**
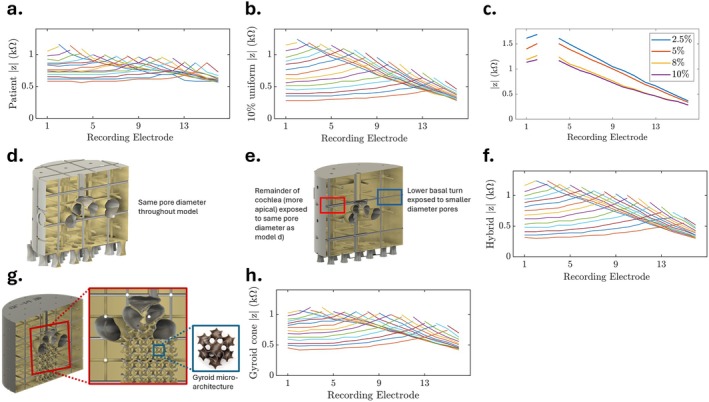
A gyroid structure with basal/apical differences in void percentages mimics the electrical characteristics of the cochlea in a 3D‐printed model. (a) Patient intra‐operative SCINSEV for reference. SCINSEVs can be recorded using clinical software and hardware and present voltages measured for all possible combinations of stimulating and recording electrodes, normalized by injected current. The morphology is representative of a typical intra‐operative SCINSEV called an EFI (electric field imaging). This was recorded using an AB 1 J CI. The same implant type was used for the following experiments. (b) Example of SCINSEV using uniform microchannel network, in this case 10% void percentage. Note the sharp drop off at the basal end, termed basal shunting. (c) Comparison of the impact of void percentage upon SCINSEVs in four different cochlea models. The anatomy was the same for each, but the void percentage was systematically adjusted. The results of one apical stimulating electrode with induced voltages recorded at all 16 CI electrodes are presented. Higher porosity leads to lower magnitude SCINSEV profiles. (d) Illustration of the uniformly distributed interlinking channels surrounding the cochlea in the 3D‐printed model. (e) Illustration of a hybrid microchannel system (featuring wider channels in the apical cochlear spiral and narrower channels in the basal region) in the 3D‐printed model. (f) Cochlear model incorporating two interlinking microchannel pore densities, whereby the basal turn was exposed to lower void percentage than the mid/apical turns. (g) Illustration of the “modiolar cone” with gyroid structure (zoomed in view). (h) SCINSEV from gyroid hybrid model, whereby a modiolar cone was created with a gyroid infill (20% void in this example). The cortical model “bone” was exposed to low density interlinking microchannels (4% void in this example), as per earlier experiments.

A network of interlinking saline channels was designed to simulate the resistive environment encountered by a CI. This was validated against voltage measurements from the CI electrode array itself, known as SCINSEVs, measurements that can routinely be rapidly obtained with clinical software and hardware, intra‐operatively and post‐operatively. They measure the voltage at the electrode array for every possible combination of stimulating and recording electrode, typically in MP mode. The interlinking saline channels were in contact with both the cochlear lumen and the surrounding saline bath. Models with varying channel architectures and porosities were fabricated to determine which bony matrix best replicated patient SCINSEV profiles (Figure 1a). Uniformly distributed interlinking channels resulted in SCINSEV profiles with a steep drop‐off toward the basal end, with higher porosity leading to lower overall SCINSEV magnitudes (Figure 1b–d). A hybrid microchannel system (featuring wider channels in the apical cochlear spiral and narrower channels in the basal region) produced more physiologically realistic SCINSEV profiles (RMSE < 0.1 kΩ along the CI) (Figure 1e,f). Further refinements to incorporate a gyroid‐based modiolar cone (Figure 1g) into the 3D‐printed cochlear models resulted in SCINSEV profiles that closely matched the morphology and shape of intraoperative patient recordings (Figure 1h). This suggests that the porous architecture of the gyroid‐based modiolar cone more accurately captures the electrochemical behavior of the natural cochlea compared to previous model designs.

**FIGURE 2:**
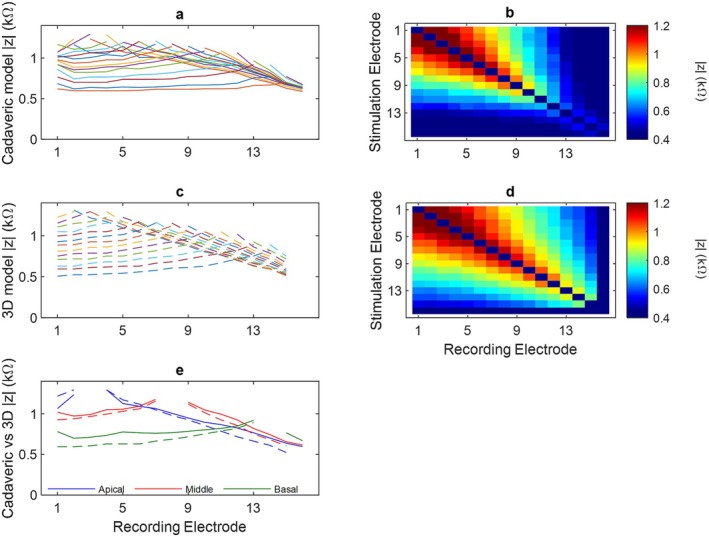
Gyroid architecture yields comparable voltage profiles between cadaveric specimens and their 3D twins. (a, b) Cadaveric SCINSEV and heatmap of SCINSEV (Cochlea C25). (c, d) 3D model SCINSEV and heatmap of SCINSEV (Cochlea C25). (e) Comparing the transimpedance arising from stimulation of an apical (blue), middle (red), and basal (green) electrode for a cadaveric cochlea (solid lines) and its equivalent 3D model (dashed lines).

SCINSEV model parameters were initially calibrated using intra‐operative measurements from five patients. Iterative adjustments were made to minimize reconstruction error (target RMSE < 0.1 kΩ) while preserving anatomical plausibility across a range of cochlear sizes (A‐values ranging from 8.4 to 9.6 mm (mean 9.0 mm)). Once consistent performance was achieved, the same model architecture was applied to simulate SCINSEVs in 12 cadaveric cochleae (Figure 2 shows one representative example; RMSE for all cochleae in Table S1).

### 2.2 Experiment 2: Comparing Complex Impedances in Cadaveric Specimens and 3D Prints

Following the successful matching of 3D “twin” models to cadaveric specimens using clinical voltage measurements, more detailed complex impedances were investigated (electrochemical impedance spectroscopy, EIS). This required research software and a custom‐built implant and was performed on six cadaveric cochleae and their 3D‐printed counterparts. Measurements were taken in triplicate for all stimulating‐recording electrode pairs (total comparisons: *n* = 768), with 45 cadaveric and 67 3D‐printed pairs excluded based on impedance magnitude and phase angle (Figure S1).

**FIGURE 3:**
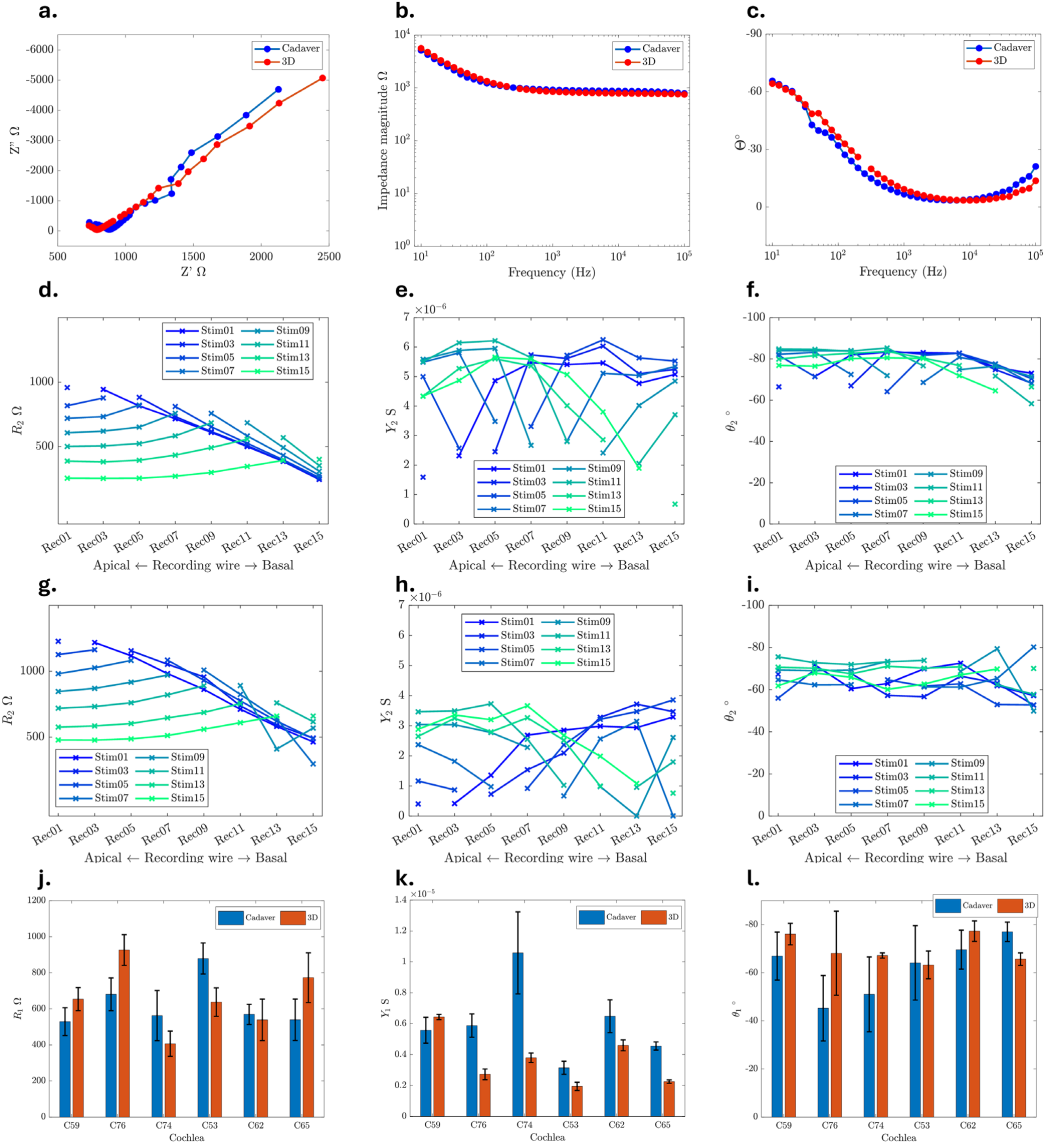
Complex impedance measurements are similar between this example cadaveric specimen and its equivalent 3D‐printed cochlear twin. (a) Nyquist plot (stimulating electrode 3, recording electrode 5). (b) Bode plot for impedance magnitude (stimulating electrode 3, recording electrode 5). (c) Bode plot for phase (stimulating electrode 3, recording electrode 5). (d) Resistor circuit 2 (R_2_): Cadaver. (e) The admittance magnitude at 1 rad/s of CPE_2_: Cadaver. (f) The constant phase of CPE_2_: Cadaver. (g) Resistor circuit 2 (R_2_): 3D. (h) The admittance magnitude at 1 rad/s of CPE_2_: 3D. (i) The constant phase of CPE_2_: 3D. (j) Mean ± standard deviation cochlear resistive component for cadaver (blue) vs. 3D twin (purple). (k) Mean ± standard deviation admittance magnitude for cadaver (blue) vs. 3D twin (purple). (l) Mean ± standard deviation constant phase component for cadaver (blue) vs. 3D twin (purple).

Bode and Nyquist plots (Figure 3a–c) demonstrate that the 3D‐printed models replicated the complex impedance profiles of cadaveric cochleae (see Figure S2 for other comparisons). In both models, the Nyquist plots demonstrate one pole, where the imaginary component becomes insignificant relative to the real component, indicating that the EIS data can be fitted using two resistor‐capacitor circuits in series. The zero‐imaginary results in the Nyquist plots corresponded to the Bode plots, where there are relatively flat curves in magnitude (Figure 3b) and near‐zero phase magnitude (Figure 3c) between 1 kHz and 10 kHz resistive behavior. These results suggest that cochlear transimpedances are influenced by different R‐CPE circuits at low and high frequencies, as has been suggested by others [22].

Despite identical cochlear geometries, the 3D‐printed models had smaller surrounding “tissue” volumes compared to the cadaveric temporal bone cubes, with potential effects on the return current pathway to ground via the NaCl bath. To isolate cochlea‐specific components, only the internal circuit components (R_2_ and CPE_2_ from the two resistor‐capacitor circuits) were compared. Morphologies of the extracted components were consistent between cadaveric cochleae (Figure 3d–f) and 3D‐printed models (Figure 3g–i), with strong agreement in mean values (Figure 3j–l). The 3D‐printed models exhibited slightly lower admittance peaks (Figure 3h) and higher R_2_ values (Figure 3g), likely due to slightly higher resistivity in the printed porous model. However, the CPE_2_ phase angle remained broadly consistent along the cochlear length in both model types, indicating that the distribution of capacitive behavior (and thus the spatial pattern of longitudinal current spread) was preserved. This consistency in phase response is important, as it reflects the balance between capacitive and resistive elements within the cochlear lumen. Taken together, the similarity in component morphology, agreement in impedance trends, and preservation of phase behavior support the use of the gyroid modiolar cone and cortical porous network. Despite minor differences in magnitude, the functional electro‐anatomical behavior of the 3D‐printed models parallels that of cadaveric tissue.

### 2.3 Experiment 3: Comparing Intra‐Modiolar Voltages in Cadaveric Specimens and 3D Prints

**FIGURE 4:**
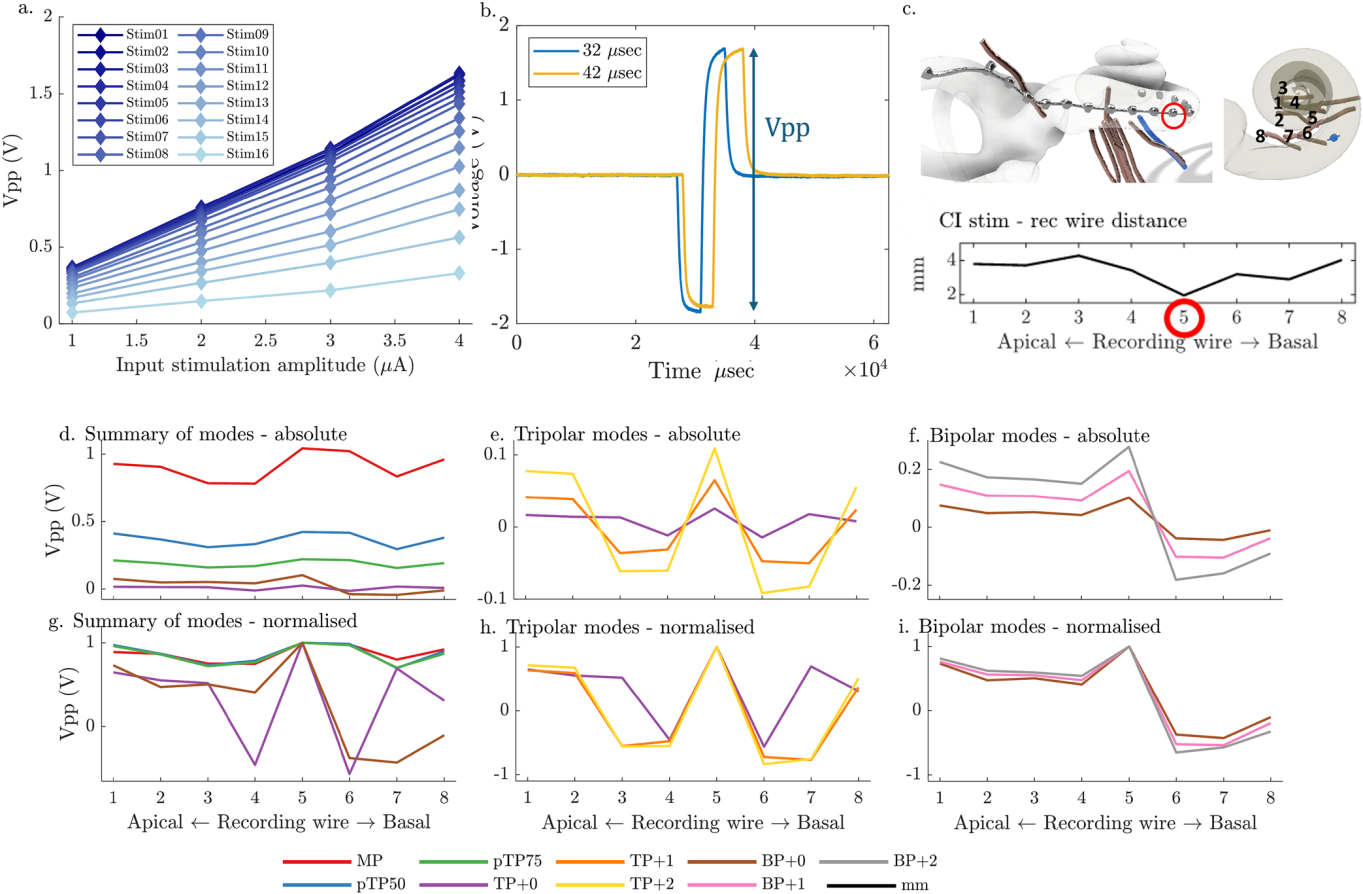
Micro‐recording wires inserted in the cadaveric models enable the measurement of voltage spread inside the modiolus, where SGNs are located. (a) Input stimulation amplitude. This was measured at recording electrode 1 from all CI stimulating electrodes 1–16 at increasing current amplitude from 500 to 2000 μA. R‐squared = 0.998. (b) Phase duration. Note the peak‐to‐peak voltage is the same irrespective of phase duration. (c) Relative position between recording wires and CI position within the cochlea. Recording wire 5 and the distance between the CI electrode closest (07) are highlighted. (d–i) Voltage measured in different recording wires for different modes of stimulation after stimulating electrode 7 on CI. The x‐axis label is the same for all subplots but is only presented for the lowermost plots for clarity. Voltage waveforms of the same polarity as the inputted waveform (cathodic‐anodic) are denoted as positive results; waveforms of the opposite polarity (anodic‐cathodic) are denoted as negative results. The peak‐to‐peak voltage (Vpp) presented on an absolute (V) and normalized scale in a cadaveric cochlea (C11) for the CI stimulating electrode (Stim07) that was closest to a mid‐basal turn recording wire, as measured under different stimulation modes. CI stimulating electrodes 14–16 were extracochlear. Monopolar (MP), partial tripolar 50%/75% (pTP50/75), tripolar (TP), bipolar (BP). Each condition was repeated 10 times. (d–g) Overview of all stimulation modes. (e–h) Tripolar+*n* modes. (f, i) Bipolar+*n* modes.

Experiment 1 and 2 measured the voltage at the level of the electrode array. Spiral ganglion neurons (SGNs) are however located inside the modiolus, which were measured with micro‐recording wires inserted inside both cadaveric and 3D‐printed models. The peak‐to‐peak voltage waveforms (Vpp) from one human cadaveric representative example are depicted in Figure 4. To ensure the linearity of the recording micro‐recording wires and system, Figure 4a,b investigate the impact of varying the stimulation current properties on the amplitude and phase duration, respectively. There was a linear relationship between the input current amplitude and measured Vpp. Phase duration did not significantly impact upon Vpp.

The relative position between the CI stimulating electrodes and micro‐recording wires are depicted in Figure 4c. Figure 4d shows Vpp for various commonly‐available stimulation modes (MP stimulation is the clinical standard, but other modes are available to try to reduce channel interactions). Under MP stimulation, voltage profiles exhibited broad distributions with peaks positioned near the stimulating electrode. Peak amplitudes in MP mode were notably higher than those observed in multipolar configurations, reflecting the extracochlear return electrodes. In contrast to MP, BP and TP stimulation modes produced lower peak voltages, with sharper profiles due to the presence of one or more flanking return contacts that restricted current flow. BP stimulation (Figure 4f,i) resulted in asymmetrical voltage distributions, with a flip in polarity toward the return electrode, while TP (Figure 4e,h) produced a more symmetric voltage decay on both apical and basal sides of the stimulating site. pTP stimulation (Figure 4d,g) demonstrated intermediate characteristics between MP and TP, with peak magnitudes reduced relative to MP but broader than those observed in fully focused TP mode, whilst the normalized curve was the same as that for MP.

**FIGURE 5:**
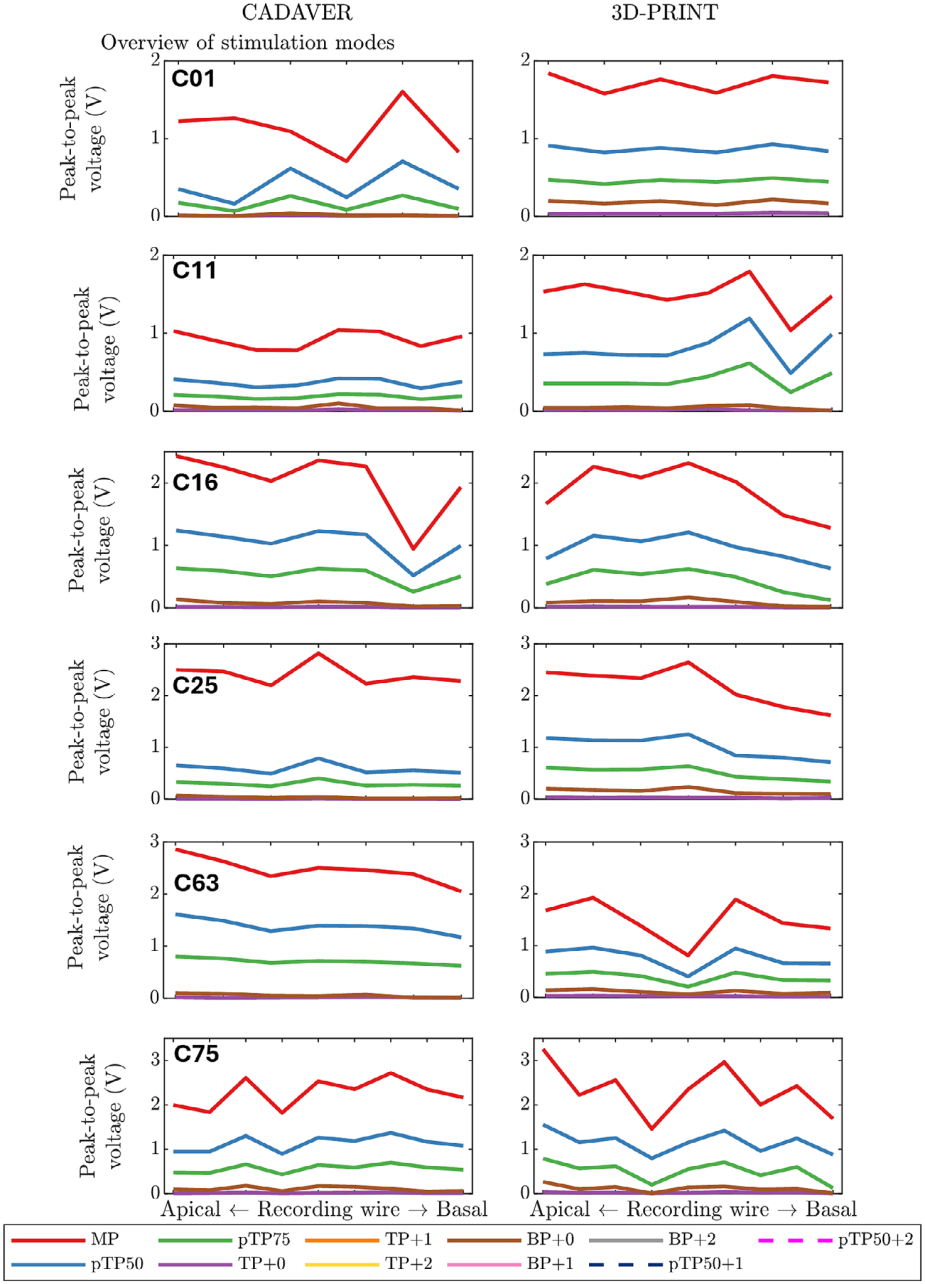
Intra‐modiolar voltage measurements are comparable between Cadaver and 3D prints. The Vpp in a cadaveric cochlea (left‐hand column) and its 3D‐printed matched pair (right‐hand column) (C75) for a mid‐array stimulating electrode (Stim08) as measured under different stimulation modes. The x‐axis is labeled the same for all subplots but is only presented for the lowermost for clarity. Monopolar (MP), partial tripolar 50%/75% (pTP50/75), tripolar (TP), bipolar (BP).

To further assess the accuracy of the 3D‐printed models in replicating human cochleae, Vpp measurements were performed on the 3D‐printed anatomical counterparts of the six cadaveric specimens. Figure 5 presents the Vpp for a mid‐array stimulating electrode in cadaveric cochleae (left column) and their 3D‐printed counterparts (right column) under different stimulation modes. Visual analysis demonstrated a strong overall agreement in Vpp trends and fluctuations across MP, TP, BP, and pTP modes. However, higher Vpp values were frequently observed in the most apically positioned wire of the 3D‐printed models. Despite these discrepancies, voltage measurements exhibited a moderate to strong correlation between cadaveric and 3D‐printed cochleae (Table 1). These findings validate the refinements made to the porous modiolar network and provide a robust basis for assessing the influence of CI array positioning and cochlear anatomy on Vpp.

**TABLE 1:** Pearson's correlation coefficient comparing Vpp for all six cochleae (cadaveric vs 3D; monopolar mode).

Cochlea	Pearson's correlation coefficient	Interpretation
C01	0.48	Moderate
C11	0.64	Strong
C16	0.54	Moderate
C25	0.65	Strong
C63	0.57	Moderate
C75	0.55	Moderate

### 2.4 Experiment 4: Impact of Array Position and Stimulation Mode on Intra‐Modiolar Voltages

Several options are available to clinicians in terms of array positioning, but its interaction with the effect of stimulation mode is yet unclear. The influence of array positioning within the cochlear lumen and multipolar mode on CI current spread was investigated for perimodiolar (PM: Cochlear CI532) and lateral wall (LW: CI522) arrays. PM arrays sit closer to the modiolus, where SGN cell bodies are located within Rosenthal's canal. To sample neural responses more uniformly and densely along the length of Rosenthal's canal, the same six cochleae used in the validation experiments were re‐printed, but with micro‐recording wires inserted through microchannels spaced at 2 mm intervals along the spiral path of the canal. This denser sampling was essential given that the physical differences between PM and LW arrays occur on relatively small spatial scales, which may not be captured adequately with sparser electrode placements.

MP was compared to BP current focusing mode. The Vpp of the 22 stimulation electrodes was measured at all micro‐recording wires for each of the 22 stimulation electrodes in six cochleae, resulting in 1166 stimulation‐recording pairs: 22 CI electrodes × 53 (5 cochlea with 9 functional micro‐recording wires +1 cochlea with 8 functional wires).

**FIGURE 6:**
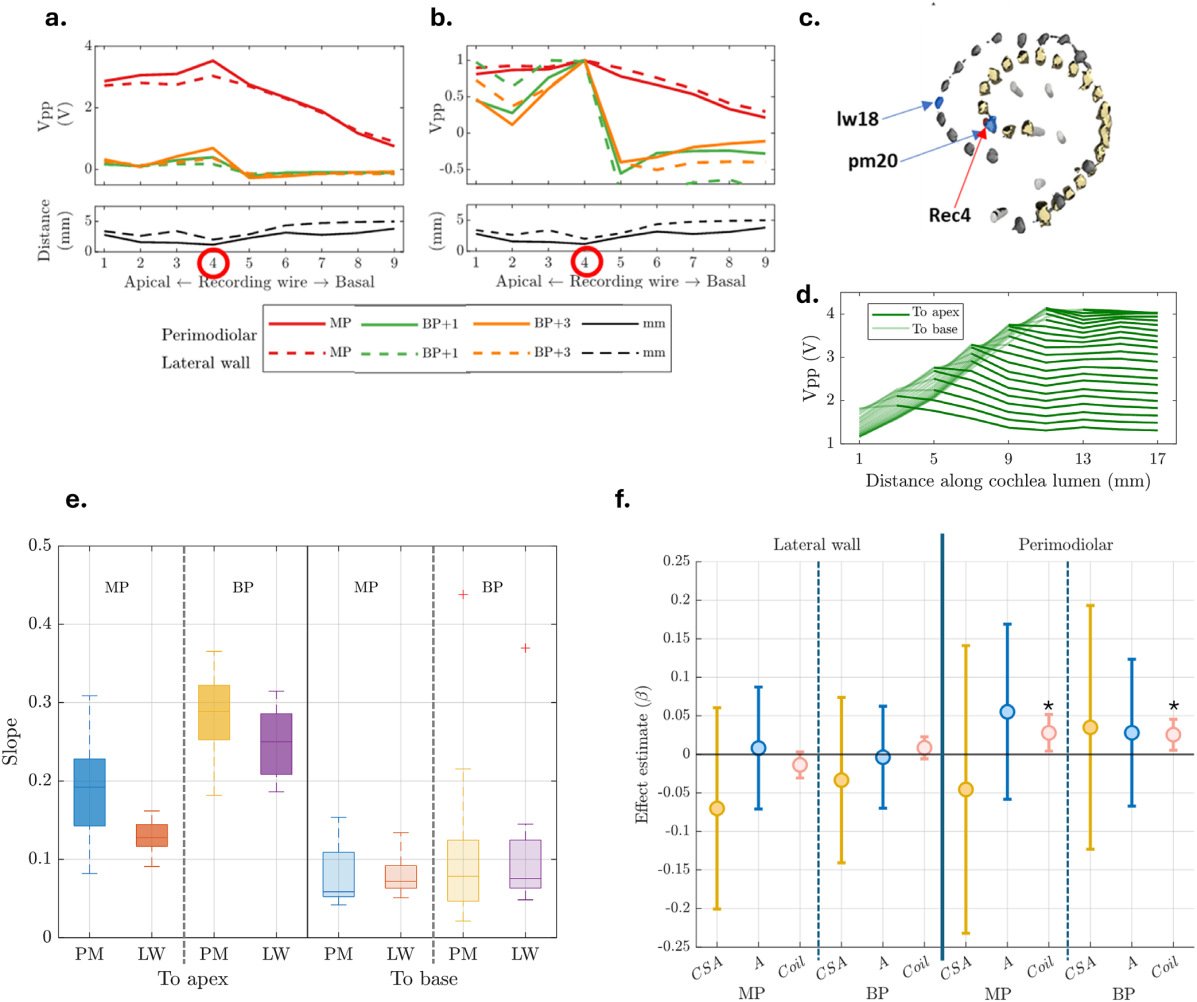
Interaction between array position and anatomy on electric field spread can be investigated in our 3D‐printed cochleae. (a) The absolute peak‐to‐peak voltage (Vpp) of one CI stimulating electrode for a perimodiolar (solid lines, electrode pm20) and lateral wall (dashed lines, electrode lw18) array, as measured in all 9 micro‐recording wires in a 3D‐printed cochlea. Pm20 and lw18 corresponded to approximately the same position in the cochlea, closest to recording wire 4 (circled on x‐axis). (b) The same data as plotted in subplot a, but on a normalized scale. (c) Relative position between recording wires and CI position within the cochlea. Recording wire 4 and the distance between the CI electrode closest (pm20, lw18) are highlighted. (d) Splitting of peak‐to‐peak voltage (Vpp) to the apex (dark green) and to the base (light green). Profiles were normalized prior to equation fitting to enable direct comparison of monopolar (MP) and bipolar (BP) fitting. (e) Boxplot of the stimulation focusing under different stimulation modes—a linear fitting was used to extract the decay parameter (the “slope”). Higher values of the slope indicate less current spread. In the boxplot—box: Interquartile range; horizontal line: Median; dashed lines: 1.5× interquartile range; dots: Outliers. Perimodiolar (PM); lateral wall (LW). (f) The estimated effect (*β* ± 95% confidence intervals) of the second linear mixed‐effects model fitting: A linear mixed‐effects model comparing the anatomy of the cochlea and array types was created (slope to the apex as no discernible difference to the base). CSA: Mean cross‐sectional area of the basal turn; A‐value: Long axis basal turn diameter; Coil: A metric to describe how tightly (or loosely) coiled the cochlea is around the modiolus. A higher coiling factor indicates a more tightly coiled cochlea. See Figure 7b. In MATLAB, the linear mixed effects model estimates the fixed effect coefficients (*β*) using generalized least squares after accounting for random effects through maximum likelihood estimation.

Figure 6a,b present example Vpp traces for a mid‐cochlear micro‐recording wire (wire 4) under different stimulation modes for PM (solid lines) and LW (dashed lines) array types. Figure 6c shows the relative anatomical positioning of the two arrays and the respective closest stimulating electrodes (PM20 and LW18). In both stimulation modes, PM arrays (positioned closer to the modiolus) produced larger Vpp responses for the same injected current.

To quantify current focusing, each Vpp profile was split into apical and basal components, as per methods in references [20, 22, 23]. In Figure 6d, the Vpp traces under MP stimulation are shown for all 22 stimulating electrodes, with dark lines indicating apical spread and light green lines representing basal spread. For each segment, the voltage decay slope was computed via linear fitting: steeper slopes indicate more rapid voltage attenuation and thus reduced current spread.

Figure 6e shows the decay slopes under MP and BP stimulation for both array types. Linear mixed‐effects (LME) modeling was used to evaluate the influence of array type and stimulation mode on slope values. Significant main effects of array type were observed in both stimulation modes. For MP stimulation, PM arrays produced steeper slopes (i.e., more focused current) than LW arrays, with an estimated increase of 0.066 (95% CI: 0.039 to 0.094), *F*(1, 258) = 22.36, *p* < 0.001. For BP stimulation, PM arrays also showed a positive effect, with an estimated increase of 0.032 (95% CI: 0.008 to 0.056), *F*(1, 225) = 6.94, *p* = 0.009. Additionally, baseline slopes were higher in BP than in MP stimulation (intercepts: BP = 0.249, 95% CI: 0.232–0.266; MP = 0.129, 95% CI: 0.109–0.148), confirming that BP mode overall results in less current spread, independent of array type.

A second LME model was used to examine the influence of anatomical variables and array type on current spread during both MP and BP stimulation. No significant main effects of any anatomical variable were observed in either stimulation mode (all *p* > 0.1). However, in both stimulation modes, a significant interaction between array type and cochlear curvature was observed: for MP, *F*(1, 252) = 5.32, *p* = 0.022; for BP, *F*(1, 219) = 6.25, *p* = 0.013, although this was not significant for MP when a Bonferroni correction was applied (*α* = 0.0167) (Figure 6e). In both cases, PM arrays showed steeper decay slopes (i.e., lower current spread) as cochlear curvature increased (MP: *β* = 0.028, 95% CI [0.004, 0.052]; BP: *β* = 0.026, 95% CI [0.005, 0.046]). These findings suggest that array proximity to the modiolus may have greater functional impact in more tightly coiled cochleae, where the curvature brings the array into closer alignment with the SGN region.

## 3 Discussion

This study demonstrates an approach for replicating the electro‐anatomic environment of the human cochlea using high‐fidelity 3D‐printed models. By integrating high‐resolution cochlear anatomy, a gyroid‐based modiolar structure, and a hybrid saline channel system, these electroanatomic twins reproduced key biophysical features relevant to CI function. The models captured patient‐level voltage spread (SCINSEV), complex impedance behavior, and stimulation‐dependent voltage distributions, offering a versatile in vitro platform to investigate CI–tissue interactions under anatomically realistic conditions.

A key innovation of this study is the integration of a gyroid lattice structure to replicate the heterogeneous resistivity of cochlear tissues. Direct validation against human cadaveric specimens, together with comprehensive electrical characterization, extends beyond the capabilities of earlier physical models that relied on uniform conductivity assumptions [19, 20]. The modiolar‐centric porosity gradient provided a practical but physiologically informed solution to mimic the anisotropic microarchitecture that shapes current flow in vivo [24]. While directly reproducing modiolar anatomy remains limited by current imaging and printing technologies, this strategy demonstrates how bioengineering approaches developed in other fields, such as bone tissue engineering [25], can be adapted to create biomimetic neural interfaces. In this instance, the gyroid lattice offered a mechanically stable yet porous medium to support precise SCINSEV control without compromising printability. This emphasis on accurately modeling the modiolus is well justified, as prior FEM studies [26, 27, 28] have demonstrated that modiolus conductivity is a critical factor in fitting electrical profiles.

Voltage recordings across stimulation modes further validated the model's ability to capture realistic intracochlear potentials. The expected differences between MP, BP, and TP configurations were recapitulated, supporting the relevance of this platform for testing both established and emerging stimulation strategies. Comparisons between perimodiolar and lateral wall electrode arrays revealed that electrode design and cochlear geometry interact dynamically to influence spatial selectivity, highlighting the importance of considering anatomical variability when evaluating device performance [19, 23].

While these electrical differences support the premise that electrode proximity enhances current focusing, the clinical implications remain nuanced. While some studies report better speech outcomes with PM arrays [29, 30, 31], others find minimal or no significant differences [32, 33, 34]. Our findings reinforce that while spatial specificity may be enhanced, differences were small and so this alone may not guarantee functional gain. Factors such as neural survival, cochlear trauma, and central processing may interact with electrical field precision to shape clinical outcomes. Thus, the benefit of closer modiolar positioning would appear to be context‐dependent and influenced by both stimulation mode and individual cochlear anatomy [35].

These observations have translational implications. First, they suggest that preoperative imaging could be leveraged more systematically [36] to quantify cochlear morphology and guide electrode choice and stimulation strategies. Although preoperative planning tools remain underutilized in clinical workflows [37, 38], integrating morphometric analysis into clinical workflows could enable a more individualized approach to programming and device selection [39, 40, 41]. Second, the ability to test stimulation paradigms in anatomically realistic, patient‐derived models opens opportunities for personalized therapy planning before or during implantation. Finally, the platform could be adapted to assess how pathological changes such as fibrosis, ossification, or congenital malformations affect current spread, offering a path toward tailoring therapy in complex surgical cases.

The study is limited by its sample size for full cadaveric‐to‐model voltage validation. However, each cochlea served as its own matched control, and extensive intra‐cochlear measurements (i.e., > 700 impedance comparisons) enhanced the robustness of comparisons. Moreover, SCINSEV profiles were validated across 17 cochleae, expanding generalisability. Nonetheless, as Vpp remains an indirect proxy for neural activation, future work should integrate neurophysiological models or direct neural recordings, potentially via organ‐on‐a‐chip platforms. This physical dataset can also inform FEMs coupled with neural excitation frameworks, enabling more physiologically grounded interpretations of the CI‐neural interface. Such models would move beyond static, resistive assumptions to incorporate the behavior observed in this study. Prior work by Frijns, Brochier, Rattay, and Bachmeier [16, 17, 26, 42] provide a strong foundation for developing and validating these coupled modeling approaches. Finally, factors such as insertion depth, electrode contact orientation, and saline conductivity may contribute to variability, despite efforts to standardize these parameters via microCT and intraoperative mapping. Full characterization of electrode array trajectory remains a technical challenge, both in vitro and clinically.

## 4 Conclusion

Looking forward, the translational potential of this platform extends beyond incremental improvements in CI programming. Physical models that combine anatomical fidelity with electrical realism can serve as testbeds for next‐generation electrode arrays, current focusing techniques, and multimodal stimulation approaches such as electro‐optical hybrids. They may also accelerate the development of computational tools by providing high‐resolution, experimentally grounded datasets for model validation. More broadly, the approach described here illustrates a framework for creating “electroanatomic twins” of other neural structures, with potential relevance for bioelectronic medicine applications ranging from retinal prostheses to vagus nerve and spinal cord stimulators. By bridging anatomy, bioelectricity, and device design, these models could help translate engineering advances into more precise, patient‐specific neurostimulation strategies.

## 5 Materials and Methods

### 5.1 Study Design

This study comprised four primary experiments designed to validate and characterize 3D‐printed cochlear models in comparison to human cadaveric specimens, and to investigate the interactions between electrode arrays, stimulation modes, and intra‐modiolar electrical recordings. The first experiment was a validation study, where 3D printing methods were refined until a good match was obtained against intra‐operative and then cadaveric recordings. Experiment 2 was a twin study, where complex impedances recorded from six 3D printed models were compared against their twin cadaveric cochleae. Experiment 3 was another twin study, this time comparing the voltage recording from six 3D printed models against their twin cadaveric cochleae. In Experiment 4, the interactions between stimulation mode and electrode arrays on micro‐electrode voltage recordings were assessed. Across all experiments, data were acquired using clinical and research recording platforms: SCINSEVs were used in Experiment 1 (measurements at the electrode array, using clinical software and hardware), Electrochemical Impedance Spectroscopy (EIS) was used in Experiment 2 (measurements at the electrode array, research hardware and software), and micro‐electrode voltage recordings in experiments 3 and 4 (located at nine matched locations in Experiment 3 and nine locations in Experiment 4, with 2‐mm spacing between each recording).

### 5.2 Micro‐Computed Tomography

In all experiments, electrode and recording wire positioning was documented using micro‐computed tomography (micro‐CT), in order to match the shape of the 3D printed models to their cochleae twins. The temporal bone specimen and 3D prints constructed from them were scanned following model preparation and immediately following all experiments to document the CI position. They were scanned using a Nikon Metrology XT‐H 225 ST micro‐CT scanner (Nikon Metrology NV, Leuven, Belgium) at 125 kV, 120 μm, 1080 projections, 2 frames per projection and 1 s exposure time. Reconstruction was at an isotropic voxel resolution of 20–30 μm.

### 5.3 Cochlear Implants

All CI insertions were performed manually by experienced researchers to simulate surgical conditions. Arrays were inserted from two manufacturers. Given the availability of intraoperative and cadaveric data, Advanced Bionics arrays were initially used (AB; Advanced Bionics LLC, Valencia, CA, USA): HiFocus 1J lateral wall array or HiFocus SlimJ (also lateral wall, but thinner). These have 16 platinum‐iridium alloy electrodes, encased within a silicone carrier. The most apical electrode is denoted as electrode 1, whilst the most basal electrode is electrode 16. The CI was connected via the AB Clarion Platinum sound processor and CPI‐2 programming interface. For the experiments to compare array location, implants from Cochlear (Cochlear Ltd., Sydney, Australia) were used, as these were the only ones with true perimodiolar locations: CI522 lateral wall array or CI532 Slim Modiolar perimodiolar array. These have 22 half‐band electrodes, encased with a silicone carrier, with two extracochlear electrodes. The most apical electrode is denoted as electrode 22, whilst the most basal electrode is electrode 1. The Cochlear CIs were connected via the Nucleus CP910 sound processor, Nucleus Freedom speech processor, and programming pod. The Cochlear Slim Modiolar arrays are inserted using a stylet, and can be re‐mounted, although they can lose some of their pre‐curved nature with subsequent insertions, thus limiting the number of possible experimental repeats.

**FIGURE 7:**
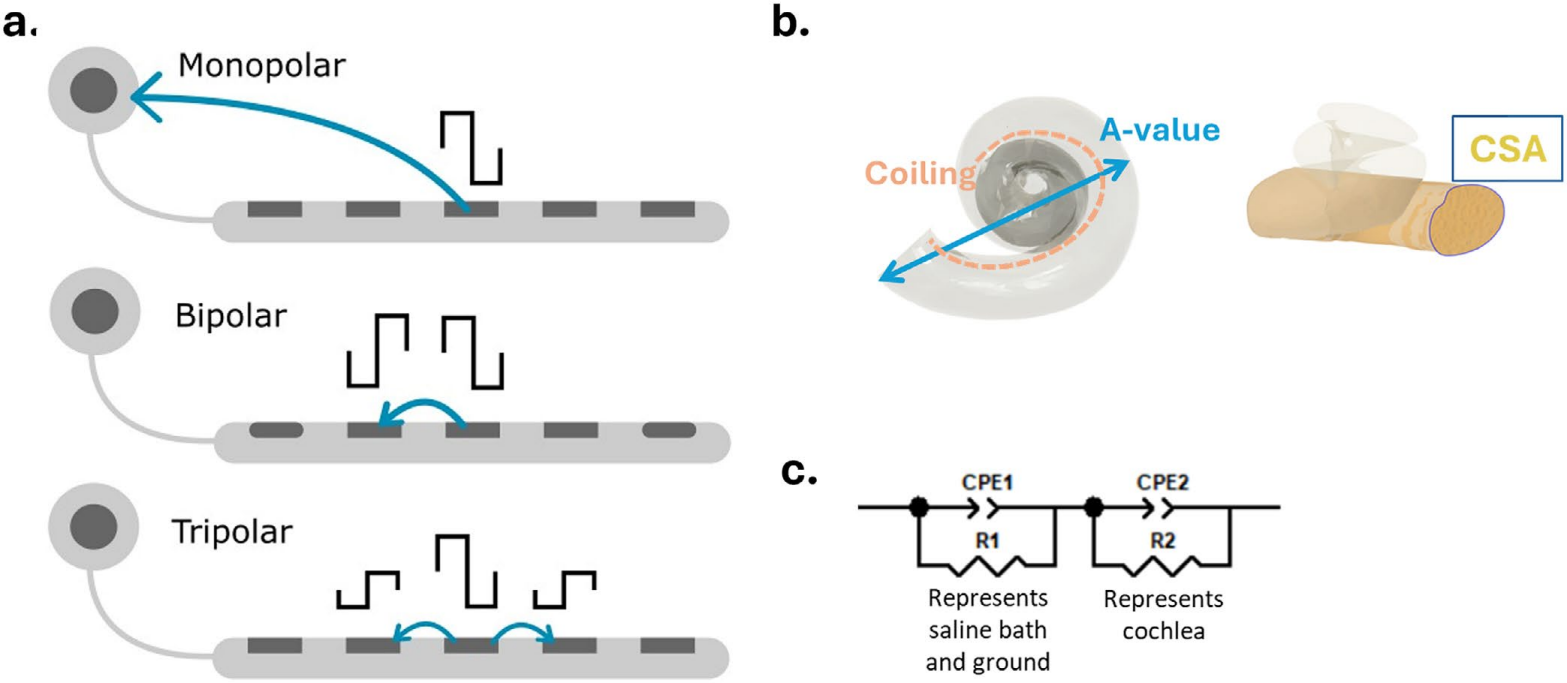
Methodological details. (a) Schematic diagram of different CI stimulation modes, with current either returning to the extra‐cochlear ground (circle) or adjacent intra‐cochlear electrodes. (b) Anatomical determinants of the cochlea. CSA: Mean cross‐sectional area of the basal turn; Coiling: A metric to describe how tightly (or loosely) coiled the cochlea is around the modiolus. A higher coiling factor indicates a more tightly coiled cochlea. (c) Schematic of equivalent circuit model. Constant phase element, CPE; Resistor, R.

Voltage waveforms were recorded under different stimulation modes: MP, BP, TP, and pTP (Figure 7a). Current was injected through an intracochlear array electrode (+I), with return directed to either intra‐ or extra‐cochlear electrodes. For MP, the return electrode is an extra‐cochlear electrode. For BP, the return electrode is an adjacent basal intra‐cochlear electrode. For TP, the return electrodes are two adjacent electrodes for half the injected current on either side. As pTP is a combination of TP and MP, the return is divided between the adjacent electrodes and the extra‐cochlear electrode.

### 5.4 Validation Datasets

The first experiment was a validation study in which 3D printing methods were iteratively refined to achieve a close match between model‐based recordings and a reference set. To ensure that the 3D‐printed models provided an accurate representation of the in vivo human cochlea, validation was first performed using real patient data before progressing to cadaveric specimens. Specifically, model development was guided by comparisons to intra‐operative SCINSEV recordings obtained from five anonymised patients, each with paired pre‐operative CT scans. The use of these retrospective clinical datasets was approved by the University of Cambridge Human Biology Research Ethics Committee (HBREC.2019.42) and the Cambridge Biomedical Research Centre (A095451). Consent was not required, as all data were fully anonymised. All patients in the reference dataset had been implanted with AB 1 J lateral wall electrode arrays.

Once the first five were refined, from a larger dataset of 83 cochleae with available microCT scans, a subset of 12 cochleae were selected to represent a wide range of anatomical variation (Table S2 and Figure S3). Selection criteria included differences in A‐value (long axis basal turn diameter), cochlear coiling (how loose or tight the cochlea is coiled around the modiolus), and cross‐sectional area (CSA) along the basal turn scala tympani (Figure 7b). These parameters were chosen as they influence array location and hence potentially electrical field distribution. By incorporating cochleae with diverse morphologies, the study aimed to ensure that the 3D printing and recording methods were robust and generalisable across the spectrum of human cochlear anatomy.

The formalin‐fixed specimens were obtained from the Human Anatomy Centre, Department of Physiology, Development and Neuroscience, University of Cambridge, HTA License Number 12146. Donors had provided written informed consent before death for use of their tissue for anatomical research in compliance with the Human Tissue Act 2004.

Formalin‐fixed temporal bone specimens were cut to approximately 4 × 4 × 4 cm^3^ and soaked in sterile 1% sodium chloride (NaCl) for 48 h, to produce tissue characteristics that were more similar to living tissue. A standard cortical mastoidectomy, posterior tympanotomy, and round window approach were performed to access the cochlea. The internal auditory meatus (IAM) was then drilled from medial (IAM side) to lateral until the falciform crest was identified. The four nerves (auditory, facial, superior vestibular, and inferior vestibular) were transected at their base, ensuring that the bony portion was not traumatized. Micro‐recording wires (200 μm thin gold wires, Alfa Aesar MFCD00003436, hand‐insulated) were inserted and secured with Gorilla Super Glue Gel (Gorilla Glue Ltd., Chorley, UK): five were inserted into the central modiolus, using a custom‐built holder, and four along the basal turn. Given that the IAM had been drilled, it was sealed off with cotton wool soaked in 1% NaCl and bone wax. The original description was a 70/30 mixture of beeswax and Vaseline [25], but this was adjusted to include a conductive electrode gel given the characteristics of the IAM: 50/30/20 mixture of Beeswax (Fisher Chemical, W/0200/50, Fisher Scientific, Belgium), Signagel Electrode Gel (Parker Laboratories Inc., 15–25, Fairfield NJ, USA), and petroleum jelly (Sigma‐Aldrich, 16415, Merck, Germany).

Immediately before implantation, a small incision was made in the round window, adjacent to the annulus, using a 21‐gauge needle. The prepared temporal bone cubes were submerged and then flushed via the round window using a micro‐catheter (MED‐EL) to remove air and debris until clear NaCl exited the round window. 1% w/v NaCl was used to mimic the electrical conductivity of perilymph [24]. To ensure that no air bubbles remained in the cochlea, the entire specimen was placed, still submerged, into a vacuum chamber until no further air bubbles extruded from the round window.

### 5.5 Designing the Electroanatomic Models

Clinical CTs of CI patients and microCTs of cadaveric cochleae were segmented using a semi‐automated workflow process in StradView (https://mi.eng.cam.ac.uk/Main/StradView) [26]. The workflow enables analysis of complex size and shape parameters. The segmented cochleae images were incorporated into a cylindrical model using a Boolean operation in Autodesk Fusion (Autodesk, USA). A 1 mm round window opening for insertion of the CI, a 1 mm in diameter apical channel to flush residual resin during post‐processing, and 300 μm microchannels for insertion of micro‐recording wires were incorporated. During Experiment 3, the microchannels were positioned in identical locations to those of the cadaveric cochleae experiments, based on microCTs of the temporal bones; for Experiment 4, they were positioned at 2 mm intervals along the modiolar axis. The porosity of the cylinder was varied to mimic in vivo electrical conductivity (see below). The models were exported as stereolithography (STL) files for printing.

The resin used for the 3D‐printed cochleae is not inherently as electrically conductive as real cochlear bone. In vivo, the petrous temporal bone, which houses the cochlea, is a dense porous structure critical for maintaining the cochlea's electrochemical environment. The cochlear modiolus has higher porosity, housing neural elements and blood vessels. Accurately replicating these porous structures in 3D printing is essential not only for anatomical fidelity, but also for achieving representative electrical behaviour, particularly as most printed cochlear models are manufactured from a single, non‐conductive material (printers with the resolution to print cochleae accurately are not multi‐material [27]). As such, conductivity must be tuned structurally, through manipulation of internal porosity filled with a conductive solution (saline) rather than by incorporating conductive materials.

Previous approaches have included connecting discrete resistors to the cochlear lumen [19] or creating homogeneous interlinking channels [20], which can be filled with saline to adjust ionic conductivity. Initially, a similar technique was used. However, these methods fell short in replicating the spatially complex, three‐dimensional ionic conduction of biological tissues. Discrete resistors yield stepwise rather than continuous variation in conductivity and rely on faradic conduction, which does not reproduce waveform smoothing or time‐dependent impedance characteristics. Homogeneous channels, while conductive, fail to reflect the spatial variation in bone porosity that shapes electrochemical behaviour in the cochlea.

To address these limitations, a hybrid porous network structure was developed, based on a gyroid infill pattern—a triply periodic minimal surface that offers both mechanical strength and physiological relevance (Figure 1g). A cone‐shaped modiolar structure was designed and printed with this gyroid pattern, reflecting the increased porosity observed along the modiolar side compared to the denser cortical walls. The gyroid architecture was selected for its high surface area‐to‐volume ratio and its capacity to disperse mechanical stress while maintaining structural integrity during the printing process. This approach aimed to provide a more biomimetic representation of the electro‐anatomical properties of the native cochlea.

### 5.6 Model Printing

Models were printed with clear microfluidic resin (V7.0a, CADworks3D μmicrofluidics, Toronto, ON, Canada) using a digital light processing (DLP) printer (CADworks3D printer M‐50, CADworks3D μmicrofluidics, Toronto, ON, Canada), with a printing area of 57 × 32 × × 120 mm and XYZ resolution of 30 μm. This printer projects a 385 nm ultraviolet wavelength through the resin to cure each layer progressively. Previous research on the accuracy of 3D printing of the human cochlea demonstrated that a DLP printer with a layer height of 30 μm yielded high model accuracy, as assessed through nominal‐actual analysis (Hrncirik 2025, under peer review). Following printing, excess resin was rinsed using isopropyl alcohol (IPA) and compressed air. Prints were cured in ultraviolet light three times for 10 s, with 1 min intervals (CureZone UV chamber, CADworks3D μmicrofluidics, Toronto, ON, Canada). Teflon‐coated copper wires (250 μm diameter) were inserted into the microchannels and secured with Gorilla Super Glue Gel.

### 5.7 SCINSEV Measurements

SCINSEV recordings can be quickly obtained with clinical software and hardware, intra‐operatively and post‐operatively. They measure the voltage at the electrode array for every possible combination of stimulating and recording electrode, typically in MP mode. They were used in Experiment 1 to rapidly test multiple models of varying porosity.

SCINSEV recordings in Advanced Bionics are known as Electric Field Imaging (EFIs), and in Cochlear are known as Transimpedance Matrix (TIM). EFIs were measured using Volta software (version 1.1.1.21032). A cathodic‐anodic MP biphasic pulse is presented, with an amplitude of 32 μA, a phase duration of 36 μs, and no interphase gap. The default stimulation mode is MP, where current flows between the active intracochlear electrode and one extracochlear electrode (built into the receiver‐stimulator). The results are reported as voltage per unit of current (kΩ).

For Cochlear, TIMs were measured using Custom Sound EP 5.1 software (version 2017). A cathodic‐anodic MP biphasic pulse was presented, with an amplitude of 105 Current Units (CU, equivalent to 117 μA), a phase duration of 25 μs, and an interphase gap of 9 μs. The default stimulation mode is MP1 + 2, where current flows between the active intracochlear electrode and two extracochlear electrodes (floating pin‐electrode and plate electrode built into the receiver‐stimulator). The results are reported as voltage per unit of current (kΩ).

### 5.8 Complex Impedances

While SCINSEVs are easily measurable in clinical settings, they offer a limited snapshot of the frequency response of the cochlea. EIS enables a more comprehensive evaluation of cochlear and surrounding tissue properties by assessing complex impedance across a spectrum of frequencies. The EIS characteristics of cadaveric and 3D‐printed models of the same cochlea were compared in Experiment 2 to ascertain whether the 3D models accurately represent the properties of cadaveric cochleae.

A custom‐built array, with the same components as a clinical SlimJ (lateral wall) array, was manufactured for these experiments by the Advanced Bionics company, with wires exposed for access. It had 16 platinum‐iridium electrodes in a silicone array, with 1 being apical and 16 being basal, and electrodes connected to pins that could then interface with a D25 connector. A ground electrode was placed in the NaCl bath, under some muscle in the cadaveric model or under the 3D print.

The methodology to perform EIS was adapted from that described by Jiang et al. [22]. In brief, after implantation, the electrode array and ground electrode were connected to two mini‐printed circuit boards (PCBs). The PCBs were connected to an impedance analyzer (RS PRO LCR‐6100), a data acquisition system (DAQ, USB‐6341, National Instruments), and power supply (Aim‐TTI EL302RD; input 12 V), using a three‐terminal configuration. The stimulating electrode was connected to the high‐force terminal, the recording electrode was connected to the high‐sense terminal, and the ground electrode was connected to both the low‐force and low‐sense terminals. To mimic components of CI clinical stimuli, the frequency range tested was from 10 Hz to 100 kHz, with 10 frequencies per decade, and an AC voltage signal with a peak amplitude of 100 mV. The order of stimulation was randomized for all combinations of alternate stimulating and recording electrodes by a custom‐built LabView programme (v2018, National Instruments Corp) and repeated in triplicate.

Results were fitted with an equivalent circuit model, which consisted of a parallel circuit of a resistor (R_1_) and constant phase element (CPE_1_) in series with another parallel circuit of a resistor (R_2_) and CPE_2_ (Figure 7c). This model was chosen to represent the cochlea electrode‐electrolyte interface (CPE_2_), resistive components of the cochlea (R_2_), the ground electrode‐electrolyte interface (CPE_1_), and resistive components of the 1% NaCl and ground (R_1_). Previous work has demonstrated that this equivalent circuit model is a good representation [22]. Data were fitted by ZView (v3.1, Scribner Associates Inc.) using complex fitting, calc‐modulus data weighting, 100 iterations, and 100 optimisation iterations. Start values were selected based on the literature and experimental values (Supporting Information and Methods S1. EIS Methodology—further details).

### 5.9 Micro‐Recording Wires Inside the Modiolus

Both methods reported record the voltage at the level of the electrode array. However, SGNs are located inside the modiolus. To assess the voltage inside the modiolus when stimulating with the CI electrode array, micro‐recording wires were therefore inserted inside the cadaveric and 3D‐printed cochleae. The inserted micro‐recording wires were connected to one mini‐printed circuit board (PCB). The experimental ground electrode was affixed to the extracochlear ground of the CI. The PCB was connected to a data acquisition system (DAQ, USB‐6341, National Instruments, USA), power supply (input 12 V), and oscilloscope (Teledyne LeCroy HDO4054A‐MS oscilloscope, sampling rate 1 GHz (19)). The order of stimulation was randomized for all combinations of stimulating CI electrode and micro‐recording wires by a custom‐built LabView programme (v2018, National Instruments Corporation), with 10 repeats for each condition.

Charge‐balanced, biphasic, cathodic‐anodic stimulation was used for both AB and Cochlear electrode arrays. For AB arrays, stimuli were delivered using Bionic Ear Data Collection System (BEDCS) research software. Stimulation consisted of 2000 μA amplitude pulses with 32 μs phase durations and no interphase gap. For Cochlear arrays, stimuli were generated in MATLAB and delivered via a Cochlear CP910 speech processor using Nucleus Implant Communicator software version 4 (NIC4). Due to platform limitations, TP mode was unavailable for Cochlear arrays; instead, BP stimulation was used as a form of current focusing. Each biphasic pulse consisted of 25 μs phase durations, a 9 μs interphase gap, and a 255 current level, delivered across a period of 120 μs. Higher‐than‐clinical stimulation amplitudes were used in both systems to enhance waveform clarity of analysis. This approach assumed system linearity across the tested amplitude range (see Results, Figure 4). Impedance values were monitored to ensure that all stimulation remained within manufacturer‐defined compliance limits. No electrodes exceeded these limits, and therefore no data were excluded.

### 5.10 Data Processing of Voltages From Micro‐Recording Wires

The peak‐to‐peak voltage (Vpp) was measured at the micro‐recording wires, which represented the neural elements anatomically in Rosenthal's canal, and imported into MATLAB. Outliers were excluded to prevent analysis of data due to broken electrodes, air bubbles, or extracochlear electrodes (Figure S4). As per convention in other publications [19, 23], waveforms with opposite polarity to those on the stimulating electrodes were denoted as negative results graphically, that is, recorded waveforms that had an anodic‐leading rather than cathodic‐leading profile.

In Experiment 3, to compare the Vpp results between the cadaveric and 3D‐printed cochleae, one mid‐array stimulating electrode (Stim08) was selected. Cadaveric and 3D‐printed results were compared visually, in terms of overall shape and trend of each model, examining for similarities or differences in the direction and slope of the lines, as well as any patterns or fluctuations. The similarity of the two models was compared using Pearson's correlation coefficient. As it was anticipated that there may be some deviations in the magnitude of Vpp, a reasonable result was defined as moderate (0.4–0.59), strong (0.6–0.79), or very strong (0.8–1) correlation. Weak correlation was specified as 0–0.39.

In Experiment 4, the spread of the electric field inside the modiolus was characterized by dividing the voltage profile into apical and basal directions. Each segment was independently fitted using a mathematical function, similar to approaches used in previous studies [19, 20, 23, 28]. Earlier work utilized exponential and power‐law models; however, these analyses had a larger number of recording points. In this study, spatial constraints within the physical spiral cochlea limited the setup to nine micro‐recording sites. Splitting the voltage profiles further reduced the number of data points available for each direction, increasing the risk of overfitting when using more complex models. Therefore, to model the voltage decay, the following linear function was used: *V*(*x*) = −λ*x + *V*_0_, where *V*(*x*) represents the voltage at distance *x* from the active electrode, *λ* is the decay slope, and *V*_0_ is the baseline voltage at the electrode.

Linear Mixed Effects (LME) models were fitted separately for MP and BP stimulation, with slope as the dependent variable. Array type (perimodiolar or lateral wall) was included as a fixed factor, and cochlear repeat was modeled as a random factor. The lateral wall configuration served as the reference array. A second LME model assessed whether anatomical parameters, including A‐value, cross‐sectional area (CSA), and cochlear curvature (Coil, where higher values indicate more tightly coiled), influenced the slope while controlling for array type. All main effects and their interactions were included as fixed factors, with cochlear repeat again specified as a random factor. *p*‐values for all significant terms and post hoc comparisons were corrected for multiple comparisons using the Bonferroni method, maintaining a significance threshold of *α* = 0.05.

## Funding

This research was supported by Wellcome Trust Developing Concept Fund (RG93172/BANCE/40181). Chloe Swords was funded by the Royal College of Surgeons of England on a Research Fellowship, and the Anatomical Society on a PhD Fellowship. Iwan Roberts was funded by the Rosetrees Trust Enterprise Fellowship (EF2020/100099), RNID Flexigrant (F112), and by the Evelyn Trust. Sita Tarini Clark was funded by the Woolf Fisher Trust, New Zealand, the Cambridge Commonwealth, European, & International Trust, and by Trinity College, University of Cambridge. Filip Hrncirik was funded by the Royal National Institute for Deaf People (RNID) Flexigrant (RNID, G100138). Professor Bance and the SENSE Lab are supported by the NIHR Cambridge Biomedical Research Centre (NIHR203312*). The views expressed are those of the authors and not necessarily those of the NIHR or the Department of Health and Social Care.

## Conflicts of Interest

M.L.B.: Decibel Therapeutics (consulting), Advanced Bionics (grant), Cochlear (grant), MED‐EL (grant), Oticon Medical (grant). All other authors declare no conflicts of interest.

## Supporting information


**Table S1:** Cochlear models incorporating a gyroid modiolar cone and interlinking cortical network, compared to intra‐operative (MC, MG, CH, MM, 2386) or cadaveric (C#) SCINSEVs for one apical, one middle, and one basal electrode Root mean square error (RMSE). Cochlea C59 was more difficult to fit at the middle electrode than the other cochleae, and required higher cortical channel porosity.
**Figure S1:** Electrochemical impedance spectroscopy The EIS and fitted equivalent circuit results are plotted for all cadavers (subplots a–c) and 3D‐printed twins (subplots d–f) are shown below. The range of the extracted parameters of the equivalent circuits was in the same range amongst all six pairs. The Nyquist plot for cochlea C53 deviates from the typical pattern in other cochleae. This may suggest the presence of air bubbles in the model or variation in a different physiological property. The extracted resistor values for the remaining five 3D‐printed twins are shown in the second figure below.
**Figure S2:** Data processing of voltages from micro‐recording wires – further details. The waveforms were normalized to remove offset introduced by environmental noise, using the *detrend* function. A low‐pass Butterworth filter (*filter* function) was applied, with a frequency of 100 kHz. The waveforms were visually inspected and those with abnormal morphology were excluded (Figure S4). When the morphology of a stimulating electrode was abnormal, that electrode was completely removed from analysis. For example, if Stim01 had abnormal morphology, Stim01 was excluded for all stimulation modes, as well as Stim04 for TP + 2 mode. Reasons for abnormal morphology may relate to reasons such as air bubbles or oxidation of the recording wire.
**Table S2:** Cochleae selected for electrochemical impedance spectroscopy (EIS) and voltage waveform peak‐to‐peak (Vpp) experiments (both cadaveric and 3D‐printed specimens). ^ST cross sectional area calculated for the mean of 1st 360 degrees, given that the majority of a CI sits in the first turn only. Standard deviation, SD; Cochlear duct length, CDL; scala tympani, ST. *The ST volume for cochlea C25 was higher than expected. A high coiling factor indicates a more tightly coiled cochlea, defined according to the cochlear radius (distance from lateral wall to modiolus).
**Figure S3:** Violin plots further visualizing the distribution of the full dataset (“All”) and experimental subsets for ST cross‐sectional area, A‐value, and coiling factor. Materials and Methods S1. EIS methodology—further details.
**Figure S4:** Example of normal and abnormal voltage waveform morphology. In this example, RecEL 4 had abnormal morphology and was excluded from analysis.

## Data Availability

The data that support the findings of this study are available from the corresponding author upon reasonable request.
