# Supplementary material for: 3D‐Printed Electroanatomic Twins of Cadaveric Cochleae: A Platform for Cochlear Implant Testing
Source: Laryngoscope Investig Otolaryngol. 2026 Jul 17;11(4):e70509. doi: 10.1002/lio2.70509 (PMC13378407; doi:10.1002/lio2.70509)
Supplement: Supplementary file 1 — Table S1: Cochlear models incorporating a gyroid modiolar cone and interlinking cortical network, compared to intra‐operative (MC, MG, CH, MM, 2386) or cadaveric (C#) SCINSEVs for one apical, one middle, and one basal electrode Root mean square error (RMSE). Cochlea C59 was more difficult to fit at the middle electrode than the other cochleae, and required higher cortical channel porosity. Figure S1: Electrochemical impedance spectroscopy The EIS and fitted equivalent circuit results are plotted for all cadavers (subplots a–c) and 3D‐printed twins (subplots d–f) are shown below. The range of the extracted parameters of the equivalent circuits was in the same range amongst all six pairs. The Nyquist plot for cochlea C53 deviates from the typical pattern in other cochleae. This may suggest the presence of air bubbles in the model or variation in a different physiological property. The extracted resistor values for the remaining five 3D‐printed twins are shown in the second figure below. Figure S2: Data processing of voltages from micro‐recording wires – further details. The waveforms were normalized to remove offset introduced by environmental noise, using the detrend function. A low‐pass Butterworth filter (filter function) was applied, with a frequency of 100 kHz. The waveforms were visually inspected and those with abnormal morphology were excluded (Figure S4). When the morphology of a stimulating electrode was abnormal, that electrode was completely removed from analysis. For example, if Stim01 had abnormal morphology, Stim01 was excluded for all stimulation modes, as well as Stim04 for TP + 2 mode. Reasons for abnormal morphology may relate to reasons such as air bubbles or oxidation of the recording wire. Table S2: Cochleae selected for electrochemical impedance spectroscopy (EIS) and voltage waveform peak‐to‐peak (Vpp) experiments (both cadaveric and 3D‐printed specimens). ^ST cross sectional area calculated for the mean of 1st 360 degrees, given that the major [file LIO2-11-e70509-s001.docx]

Supplementary Information

|  | Model characteristics | | Apical electrode | Middle electrode | Basal electrode |
| --- | --- | --- | --- | --- | --- |
| Cochlea | Cortical channel fraction % | Gyroid fraction % | RMSE | RMSE | RMSE |
| MC | 4 | 20 | 0.08 | 0.04 | 0.08 |
| MG | 3.3 | 20 | 0.09 | 0.08 | 0.09 |
| CH | 3.3 | 20 | 0.07 | 0.04 | 0.07 |
| MM | 3.3 | 20 | 0.08 | 0.06 | 0.07 |
| 2386 | 3.3 | 20 | 0.08 | 0.05 | 0.06 |
| C01 | 4 | 20 | 0.09 | 0.04 | 0.04 |
| C11 | 4 | 30 | 0.09 | 0.09 | 0.08 |
| C16 | 4 | 15 | 0.03 | 0.05 | 0.09 |
| C25 | 3.3 | 10 | 0.07 | 0.05 | 0.09 |
| C53 | 4 | 30 | 0.9 | 0.06 | 0.06 |
| C59 | 9* | 20 | 0.09 | 0.14* | 0.03 |
| C62 | 3.3 | 20 | 0.09 | 0.09 | 0.05 |
| C63 | 3.3 | 15 | 0.09 | 0.07 | 0.09 |
| C65 | 3.3 | 15 | 0.1 | 0.08 | 0.1 |
| C75 | 3.3 | 10 | 0.1 | 0.07 | 0.09 |
| C74 | 4 | 20 | 0.09 | 0.1 | 0.09 |
| C76 | 3.3 | 15 | 0.07 | 0.1 | 0.05 |

Table S1 Cochlear models incorporating a gyroid modiolar cone and interlinking cortical network, compared to intra-operative (MC, MG, CH, MM, 2386) or cadaveric (C#) SCINSEVs for one apical, one middle, and one basal electrode Root mean square error (RMSE). Cochlea C59 was more difficult to fit at the middle electrode than the other cochleae, and required higher cortical channel porosity.


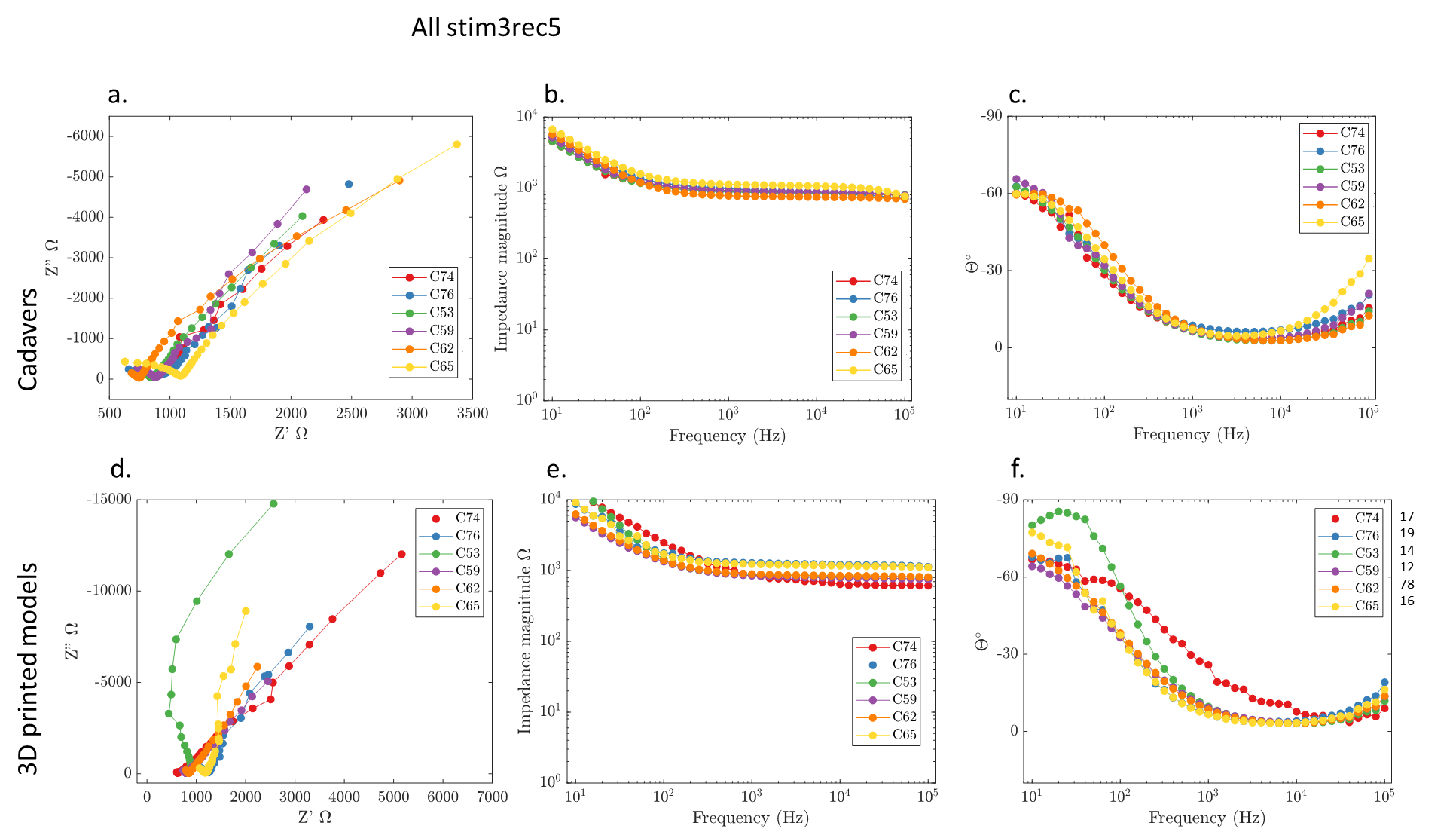


Fig. S1 Electrochemical impedance spectroscopy The EIS and fitted equivalent circuit results are plotted for all cadavers (subplots a-c) and 3D-printed twins (subplots d-f) are shown below. The range of the extracted parameters of the equivalent circuits was in the same range amongst all six pairs. The Nyquist plot for cochlea C53 deviates from the typical pattern in other cochleae. This may suggest the presence of air bubbles in the model or variation in a different physiological property. The extracted resistor values for the remaining five 3D-printed twins are shown in the second figure below.


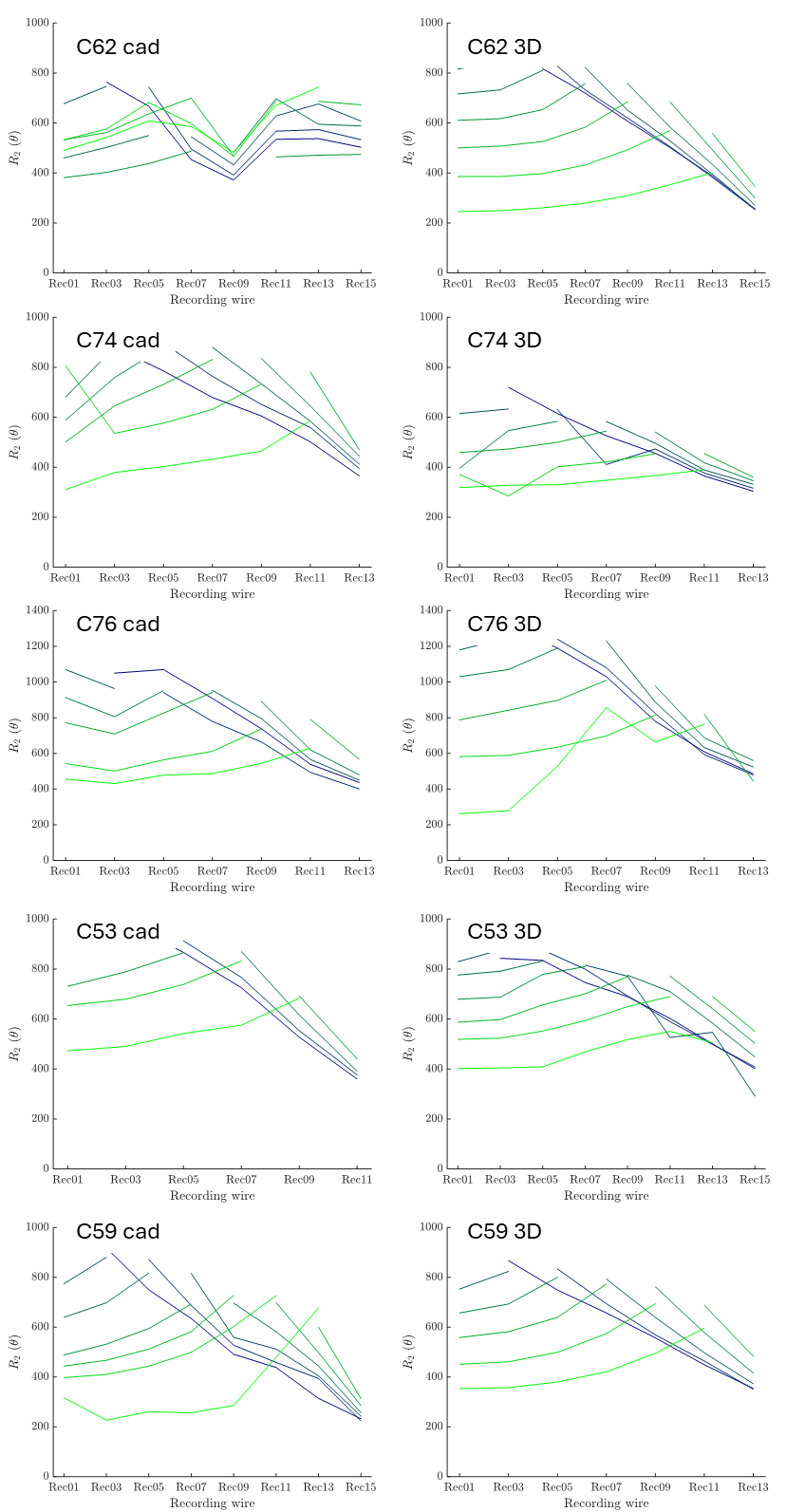


Fig. S2 Data processing of voltages from micro-recording wires – further details The waveforms were normalised to remove offset introduced by environmental noise, using the *detrend* function. A low-pass Butterworth filter (*filter* function) was applied, with a frequency of 100 kHz. The waveforms were visually inspected and those with abnormal morphology were excluded (Supplementary Material Figure S4). When the morphology of a stimulating electrode was abnormal, that electrode was completely removed from analysis. For example, if Stim01 had abnormal morphology, Stim01 was excluded for all stimulation modes, as well as Stim04 for TP+2 mode. Reasons for abnormal morphology may relate to reasons such as air bubbles or oxidation of the recording wire.

| **Cochlea #** | **Experiment** | **ST cross sectional area^** | **A-value (mm)** | **Coiling factor** |
| --- | --- | --- | --- | --- |
| Mean±SD (n=83) | - | 1.68 (0.33) | 8.95 (0.38) | 9.76 (1.61) |
| C01 | Vpp | 1.72 | 9.6 | 10.4 |
| C11 | Vpp | 1.69 | 9.5 | 7.9 |
| C16 | Vpp | 1.87 | 9.6 | 8.4 |
| C25 | Vpp | 1.67 | 8.8 | 9.2 |
| C63 | Vpp | 1.38 | 8.9 | 10.7 |
| C75 | Vpp | 1.25 | 8.9 | 7.8 |
| C74 | EIS | 1.80 | 9.4 | 11.6 |
| C53 | EIS | 1.77 | 9.4 | 7.3 |
| C62 | EIS | 1.75 | 9.3 | 11.6 |
| C76 | EIS | 1.23 | 8.4 | 10.9 |
| C59 | EIS | 1.57 | 8.7 | 10.8 |
| C65 | EIS | 1.46 | 8.7 | 11.1 |

Table S2. Cochleae selected for electrochemical impedance spectroscopy (EIS) and voltage waveform peak-to-peak (Vpp) experiments (both cadaveric and 3D-printed specimens) ^ST cross sectional area calculated for the mean of 1^st^ 360 degrees, given that the majority of a CI sits in the first turn only. Standard deviation, SD; Cochlear duct length, CDL; scala tympani, ST. *The ST volume for cochlea C25 was higher than expected. A high coiling factor indicates a more tightly coiled cochlea, defined according to the cochlear radius (distance from lateral wall to modiolus).


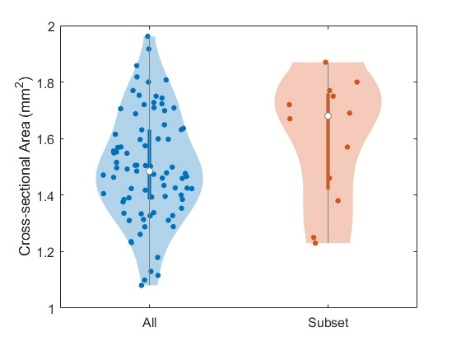

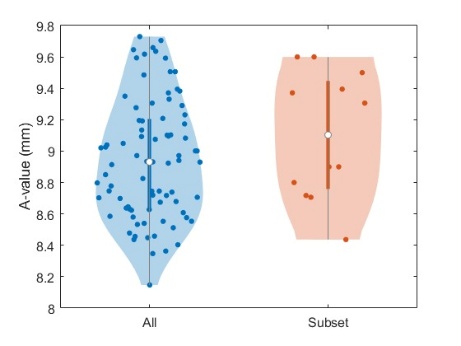

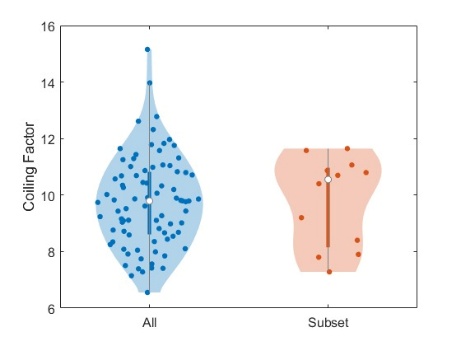


**Fig. S3 Violin plots further visualising the distribution of the full dataset (‘All’) and experimental subsets for ST cross-sectional area, A-value, and coiling factor.**

Materials and Methods S1. EIS methodology – further details

EIS outliers were identified for individual electrodes when the phase angle was more than three standard deviations from the mean phase angle across repeats, the mean absolute impedance magnitude (|Z| in Ω) was higher than 50 kΩ at 10 Hz, or the mean phase angle was greater than -5^o^ degrees at any point from 10 Hz and 1 kHz. The remaining analyses were performed with the mean values. The start values for fitting in Zview were defined as follows: the resistors (R_1_ and R_2_) were defined as the absolute impedance magnitude as the phase approached 0^o^ (1E6 and 1000, respectively). The start values of the constant phases of the CPE were defined as 0.8 and 0.9 for CPE1-P and CPE2-P, respectively. The start values of the admittance magnitude were defined as 1E-8 and 1E-6, respectively. No values were constrained.


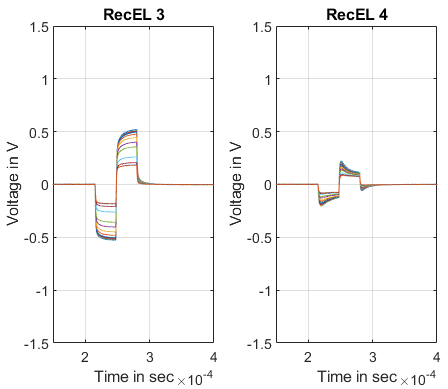


Fig. S4 Example of normal and abnormal voltage waveform morphology

In this example, RecEL 4 had abnormal morphology and was excluded from analysis.
